# A Case Report of McArdle Disease Diagnosed Following Statin-Induced Myositis

**DOI:** 10.7759/cureus.44701

**Published:** 2023-09-05

**Authors:** João S Teles, Catarina T Ramos, Beatriz M Almeida, Anabela V Sousa

**Affiliations:** 1 Family Medicine, Unidade de Saúde Familiar (USF) Brás-Oleiro, Agrupamento de Centros de Saúde (ACeS) Gondomar, Porto, PRT

**Keywords:** exercise intolerance, statins, myalgias, myophosphorylase, mcardle disease

## Abstract

McArdle disease is a rare condition, characterized by a deficiency of phosphorylase muscle isoform, an enzyme responsible for the breaking down of glycogen, necessary for obtaining energy. Patients typically present with exercise intolerance, myalgias, fatigue, cramps, muscle stiffness, and/or weakness induced by physical activity. The diagnosis is generally established late, with a median delay of about 29 years. We present the case of a female patient with a long history of myalgias, muscle weakness, and exercise intolerance, diagnosed with McArdle disease by the age of 74, after statin-induced myopathy. We aim to review the diagnosis and treatment of this disease, as a way to raise awareness among the medical community.

## Introduction

McArdle disease, also known as myophosphorylase deficiency, is a rare condition (with an estimated prevalence of 1 in 100,000-140,000 patients, although the exact prevalence is not known) caused by a deficiency of muscle isoform of phosphorylase (PYGM). It is an autosomal-recessive disorder, meaning that its manifestation is dependent on pathogenic variants of both alleles of the *PYGM* gene. This particular enzyme is responsible for the breaking down of glycogen in muscle cells, necessary for obtaining energy. Therefore, its absence impacts the normal function of muscle cells, especially during physical activity [1-3]. Patients typically present with exercise intolerance, myalgias, fatigue, cramps, muscle stiffness, and/ or weakness that can be induced by isometric (e.g., weightlifting) or dynamic (e.g., jogging) exercises. Symptoms are usually present during the first decade of life but appear to be more prominent during adolescence or early adulthood [4]. Given its rarity, the diagnosis is often delayed by a median of about 29 years [5].

We present the case of a female patient with a long history of myalgias, muscle weakness, and exercise intolerance, diagnosed with McArdle disease by the age of 74, after statin-induced myopathy. The main goal of this clinical case is to raise awareness about this condition to allow an earlier diagnosis. We also aim to review its diagnosis and treatment.

## Case presentation

In 2016, a 68-year-old Portuguese woman (from Penafiel), with a medical history of being overweight, was consulted by her family doctor on the account of low back pain accompanied by aches located in both thighs, precipitated by physical activity. The physical examination of this patient was normal, and she was submitted to a lumbosacral spine computed tomography (CT) scan to look for lumbar radiculopathy. The results showed L4/L5 level disc protrusion and facet hypertrophy, leaving doubts on whether there was radicular compression. A referral was made to the neurosurgery specialty, and a further study with magnetic resonance imaging (MRI) excluded existing nerve compression. It was assumed to be a case of pain due to lumbar degenerative disease, and the patient was treated with a short course of a systemic low-dose non-steroidal anti-inflammatory drug and advised to lose weight and seek physiotherapy as well as hydro gymnastics. Despite the treatment, the patient was still symptomatic and consulted with her family medicine practitioner once more, who was unable to explain the thigh ache.

In 2019, the same patient was diagnosed with dyslipidemia and medicated by her family doctor with atorvastatin 20 mg. Six months after starting this new drug, the patient revealed worsening of the myalgias located in both thighs. The following tests to which she was subjected revealed an elevated creatine kinase (CK) level (433 U/L). Atorvastatin 20 mg was suspended, and pitavastatin 2 mg was started instead. After one month, the myalgias were still present and the CK value was even higher (1087 U/L). Her family doctor made the diagnosis of statin-induced myopathy, and pitavastatin was stopped. Two months later, although the patient reported a slight improvement in her myalgias since the discontinuation of statin therapy, she still presented with significant pain, especially during physical activity. CK value had decreased but was still high (CK 747 U/L). The patient was then referred to the Rheumatology care for further examination. 

In the first rheumatology appointment, the patient revealed that she had been experiencing myalgia and muscular weakness in both thighs since childhood, particularly when engaging in physical activities such as brisk walking, running, or even jumping. She always needed to take short breaks to rest, which appeared to alleviate her symptoms. Because of this, she was known by her family and friends to be a *lazy person*. No doctor was ever capable of explaining the source of her pain, and no medicine was effective.

Throughout all the consultations with her rheumatology physician, the physical examination did not reveal any signs of muscle weakness in either her upper or lower limbs, and she demonstrated no limitations in her ability to rise from a chair. No other anomalies were noted. CK values remained persistently high (566 U/L >> 480 U/L >> 860 U/L), and the results of other muscular enzymes were also abnormally high: lactate dehydrogenase (LDH) (355 U/L >> 297 U/L >> 302 U/L), aldolase (13.4 U/L >> 12.8 U/L >> 14.3 U/L), and myoglobin (256.2 ng/mL >> 185 ng/mL >> 316 ng/mL). Despite this, myoglobinuria was not present, and renal function was normal. A panel of antibodies usually present in inflammatory myopathies was run and anti-nuclear antibody was also tested, with negative results. 

**Figure 1:**
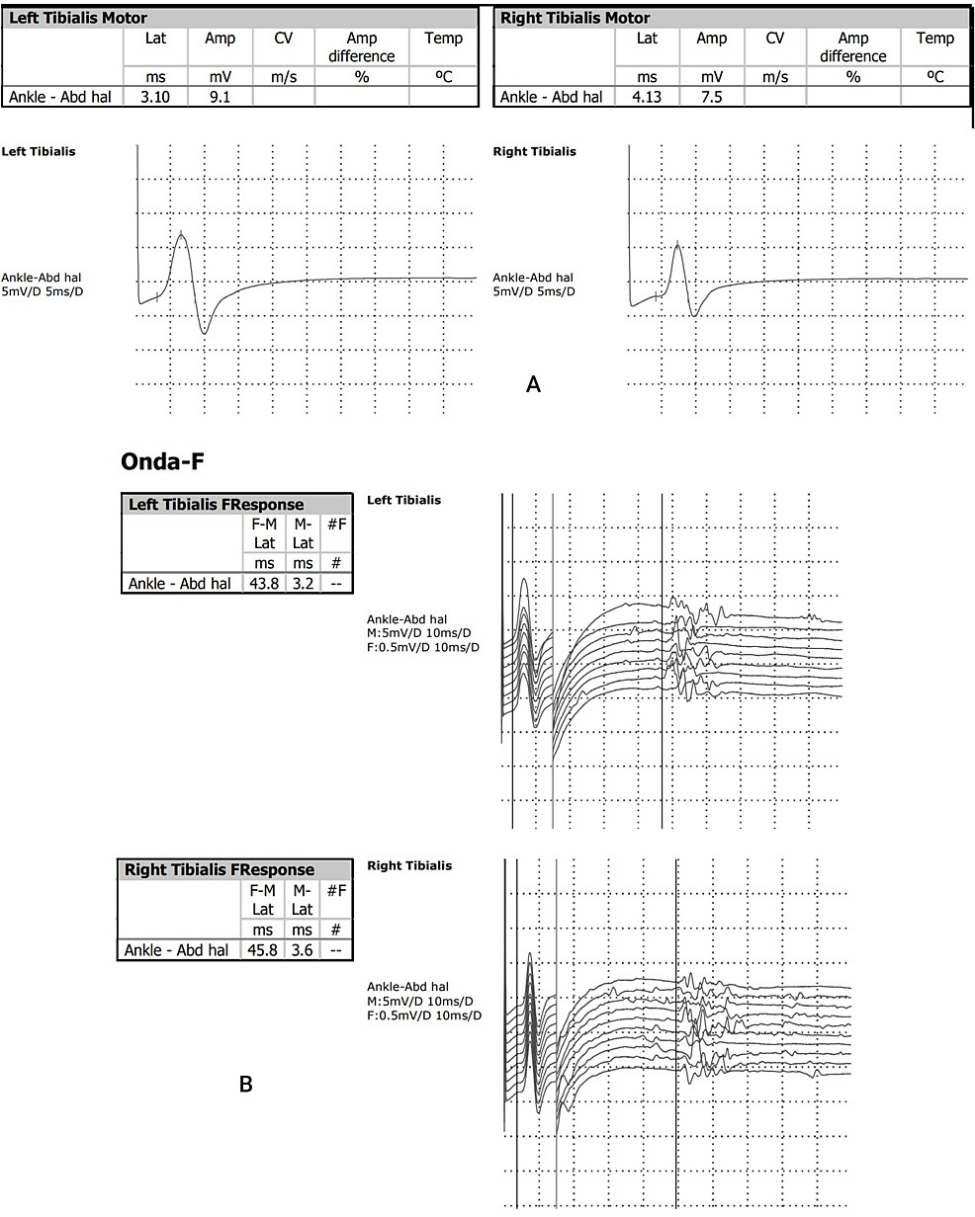
Results of electromyography. (A) Motor nerve conduction study of tibial nerve; (B) tibial F wave. Electromyography shows features of myopathic disease in the proximal muscles of the left leg, as well as in the distal muscles of both legs.

Electromyography of the lower limbs revealed features of myopathic disease in the proximal muscles of the left leg, as well as the distal muscles of both legs (Figure 1).

Sensitive abnormalities were not detected. The patient was then submitted to a biopsy of the tibialis anterior muscle, and the results showed numerous muscular fibers with subsarcolemmal glycogen vacuolation and a negative histochemical staining for myophosphorylase, in alignment with the diagnosis of McArdle disease. The patient was referred to genetic consultation, and the bidirectional and direct sequencing of the *PYGM* gene was conducted, detecting two variants in heterozygosity: c.2083G>A p.(Gly695Arg), classified as probably pathogenic, and c.2392T>C p.(Trp798Arg), classified as pathogenic. The genetic study of her two sons is still pending to confirm compound heterozygosity in the patient. This was a particularly late diagnosis of McArdle disease at the age of 74. After genetic testing was conducted, the patient revealed she had a son and a sister with similar complaints since childhood. It is relevant to mention that there is no history of consanguinity between the patient's parents or between her and her husband. 

The patient was advised to follow a carbohydrate-rich diet, including the consumption of simple carbohydrates before engaging in physical activity. Additionally, she was recommended to engage in regular aerobic exercise of light to moderate intensity while avoiding strenuous workouts. She was also referred to a nutrition consultation. 

## Discussion

Myophosphorylase is an enzyme present in muscle cells that catalyzes the degradation of glycogen into glucose-1-phosphate, necessary for cells to obtain energy. Deficiency in this enzyme leads to glycogen accumulation and an inability to generate energy from this substrate. Myophosphorylase deficiency (also known as McArdle disease) is an autosomal recessive disorder caused by pathogenic variants in both alleles of the myophosphorylase gene (*PYGM* gene, located at 11q13). Carriers of this disease (carrying a pathogenic variant in only one allele) are asymptomatic [1-3]. The patient presented in this clinical case had different pathogenic variants in both alleles that have been previously described. Several pathogenic variants of this gene have been described, but no genotype-phenotype correlations have been established [6]. The phenotype can even be modulated by polymorphic variants of other genes, such as angiotensin-converting enzyme locus. Because of this, one study suggested that treatment with angiotensin-converting enzyme inhibitor ramipril could lead to an improvement in symptoms [7].

Patients typically present with exercise intolerance beginning in infancy, adolescence, or early adulthood. Because their muscle cells are not capable of obtaining energy from glycogen metabolism, patients frequently suffer from myalgia, muscle rigidity, and/ or weakness, particularly during physical activity and alleviating with rest. A *second wind* phenomenon is also described, characterized by a temporary marked improvement after about 10 minutes of exercise. This can be explained by increased blood flow, enhanced delivery of free fatty acids to muscle cells (which function as an energy source), as well as utilization of glucose hepatic storage [4]. McArdle disease is not life-threatening, although there is a risk of rhabdomyolysis and myoglobinuria, particularly after intense or prolonged exercise. Myoglobinuria can then lead to acute kidney injury, requiring emergent treatment [8]. Another typical feature of this condition is the persisting high levels of CK, either after physical exertion or even during rest. The presence of recurrent exercise intolerance and/ or persisting elevated levels of CK should raise suspicion about the diagnosis of McArdle disease [8].

Given its low prevalence and clinicians’ overall lack of knowledge about this disease, misdiagnosis is common. Many times, patients are diagnosed with other rheumatic or neuromuscular diseases and even psychological conditions. Other times, people affected by this condition are simply considered to be *unfit* or *lazy*. If the complaints start during childhood, it is common to assume it’s just *growing pain*. As a result, there is an average diagnostic delay of 29 years, and patients may endure many years without a clinical explanation for their symptoms and persistent complaints, even when undergoing treatment [5]. In this clinical case, the woman was first treated with physical rehabilitation and pain medication, without any clear symptom improvement. A history of myalgias and exercise intolerance was present since childhood, which should have raised suspicion of her actual condition. Furthermore, the patient had similar cases in her family, which further suggests the presence of a metabolic myopathy. The awareness of this disease, as well as a complete clinical history, is essential for a correct diagnosis. In this case, it could have led to an earlier diagnosis.

A variety of tests can be helpful to establish the diagnosis of McArdle disease. Genetic testing through either sequencing of *PYGM* or sequencing panels for several myopathies, including myophosphorylase deficiency, is the most efficient and least invasive means of confirming the diagnosis. If the clinical history is not clear, a forearm semi-ischemic test or muscle biopsy with biochemical or histochemical analysis (that includes testing for myophosphorylase) can be performed. In the case of an abnormal result, genetic testing is necessary. The preferred forearm exercise test is a nonischemic or minimally ischemic test, which consists of having the patient perform one-second-hand grips, with maximal effort, every other second for one minute, while blood samples are taken [9]. On the other hand, muscle biopsy, an invasive test, when executed with histochemical staining for myophosphorylase, can be helpful for diagnosis, since it typically reveals a lack of activity of this enzyme in muscle fibers and may also reveal focal subsarcolemmal and intermyofibrillar accumulations of normally structured glycogen [10,11]. 

Several cases of myopathy induced by statins have been reported in patients affected by McArdle disease, some resulting in rhabdomyolysis [12]. This way, statin therapy may lead to worsening of symptoms of myalgia and exercise intolerance in patients with this disease. In undiagnosed patients, this can eventually contribute to establishing the diagnosis, as represented by this clinical case. After the withdrawal of the statin, the CK value should return to normal range values. If this does not happen, it could be a sign of a metabolic myopathy, such as myophosphorylase deficiency. In this clinical case, despite atorvastatin being replaced by pitavastatin and later withdrawal of pitavastatin, CK values remained high and the patient was still symptomatic. Ultimately, this led to the clinical suspicion of a metabolic myopathy and a later diagnosis of McArdle disease. In diagnosed patients, the treatment of comorbid dyslipidemia is challenging and statins should be used with caution. Statins such as fluvastatin, pravastatin, or pitavastatin are preferred, as they are less likely to cause myopathy and other musculoskeletal symptoms [13].

Even though several therapies have been studied for this disease, there is still a general lack of knowledge on this topic. Most effective treatments include dietary changes and gradually applied moderate physical activity. A higher carbohydrate diet may improve patients' symptoms, as it allows maintenance of hepatic glycogen stores and further mobilization of hepatic glucose during physical activity [14,15]. Ingestion of simple carbohydrates such as sucrose (e.g., sports drinks) before exercising may improve tolerance, especially during aerobic exercise [16]. On the other hand, previous consumption of other fast-acting simple carbohydrates, such as glucose and fructose, does not seem to be beneficial in these patients [15]. Regular practice of light-to-moderate intensity aerobic physical exercise, such as walking or cycling, has been shown to have benefits in terms of exercise tolerance [17].

## Conclusions

This clinical case portrays a patient with typical complaints since childhood, in which the diagnosis was only established very late in life. A detailed clinical history could have made a difference and allowed an earlier diagnosis. McArdle disease is rare and should be suspected if the patient presents with a long history of exercise intolerance, myalgias and muscular weakness, and/or rigidity caused by physical activity, as well as persistently high levels of CK (which can be evident after discontinuation of a statin). A familiar history of similar complaints, although rare, can also be present, which raises even more suspicion of the disease. Raising awareness of the existence of McArdle disease is crucial to achieving earlier diagnosis and, consequently, alleviating patients' suffering.
